# Survey on computer aided decision support for diagnosis of celiac disease

**DOI:** 10.1016/j.compbiomed.2015.02.007

**Published:** 2015-10-01

**Authors:** Sebastian Hegenbart, Andreas Uhl, Andreas Vécsei

**Affiliations:** a Department of Computer Sciences, University of Salzburg, Jakob-Haringer Strasse, 5020 Salzburg, Austria; b St. Anna Children׳s Hospital, Medical University Vienna, 1090 Vienna, Austria

**Keywords:** Computer, Automated, Assisted, Diagnosis, Celiac, Disease, Endoscopy

## Abstract

Celiac disease (CD) is a complex autoimmune disorder in genetically predisposed individuals of all age groups triggered by the ingestion of food containing gluten. A reliable diagnosis is of high interest in view of embarking on a strict gluten-free diet, which is the CD treatment modality of first choice. The gold standard for diagnosis of CD is currently based on a histological confirmation of serology, using biopsies performed during upper endoscopy. Computer aided decision support is an emerging option in medicine and endoscopy in particular. Such systems could potentially save costs and manpower while simultaneously increasing the safety of the procedure. Research focused on computer-assisted systems in the context of automated diagnosis of CD has started in 2008. Since then, over 40 publications on the topic have appeared. In this context, data from classical flexible endoscopy as well as wireless capsule endoscopy (WCE) and confocal laser endomicrosopy (CLE) has been used. In this survey paper, we try to give a comprehensive overview of the research focused on computer-assisted diagnosis of CD.

## Highlights

• The state-of-the-art research in automated diagnosis of celiac disease is presented.
• A systematic review of methods and techniques used in this field is given.
• Specific issues and challenges in the field are identified and discussed.

## 1 Introduction

Celiac disease (CD) is a multisystemic immune-mediated disease, which is associated with considerable morbidity and mortality [4,44,82]. The prevalence of CD in Europe and North America ranges from 1:80 to 1:300 [11,12,28,29,32,61,66]. In the pathogenesis of CD, a pivotal contribution is made by a dysregulated immune response directed against tissue transglutaminase type 2 (TG2) which has been identified as a prominent autoantigen of CD [27]. The exogenous trigger of this dysregulated immune response is gluten which is ingested along with grain-containing food. Gluten is the main protein of specific grains like wheat, barley and rye.

CD displays itself through a wide spectrum of clinical manifestations including gastrointestinal symptoms such as abdominal pain, chronic diarrhea, bloating, nausea and vomiting as well as more general features like failure to thrive, weight loss and fatigue. There are also numerous extraintestinal CD manifestations like dermatitis herpetiformis [6,97], alopecia areata and cerebellar ataxia [47] and various other neurologic and psychiatric diseases, iron deficiency [65] and premature osteopenia. It has been estimated that seven out of eight patients with CD remain undiagnosed because most cases of CD only have minor gastrointestinal symptoms [13].

In untreated or inappropriately treated CD the inflammation caused by the dysregulated immune response can disrupt the intestinal mucosa thus leading to a total atrophy of the villi, which are finger-like projections of the mucosa. After embarking on a strict gluten-free diet (GFD), which is the CD treatment modality of first choice, the inflammation gradually subsides allowing for mucosal healing. Strict adherence to a GFD for life is required if acute and chronic complications are to be avoided [88]. To avoid the most severe complications of CD, early and reliable diagnosis of CD for commencing a strict GFD is of vital importance.

Besides for a small proportion of children fulfilling certain clinical, serological and genetic criteria [64], biopsy of the small intestine remains the gold standard for CD diagnosis and is usually recommended as CD requires a lifelong commitment to a strict GFD.

The mucosal alterations caused by CD are classified into different stages of severity. Oberhuber [77] modified a widely used staging of severity by Marsh [75] in 1999. This histological classification scheme identifies six classes, ranging from class Marsh-0 (no visible change of villi structure) up to class Marsh-3C (absent villi).

Unfortunately, the histological staging of biopsies is subject to a significant degree of intra- and inter-observer variability [76,1,85,96,23]. Therefore, observer independent diagnostic methods such as computer-assisted diagnosis systems are urgently needed. Furthermore, the whole diagnostic work-up of CD, including duodenoscopy with biopsies, is time-consuming, cost-intensive, and rather invasive. Consequently, to save costs, time, and manpower and at the same time increase the safety of the procedure, a less invasive approach avoiding biopsies would be highly desirable. Recent studies by Cammarota et al. [10,8] investigating such endoscopic techniques report reliable results. Current diagnostic methods are entirely observer-dependent and require significant knowledge, expertise, and time.

An interesting field where computer-assisted video analysis could be useful is the application within follow-up endoscopies of celiac patients on a GFD, testing if a re-growth of villi has taken place. Another advantage of computer-assisted video analysis is quality improvement rendered possible by means of telemedicine, for example, by obtaining a second opinion after endoscopic video clips have been transmitted to institutions that have video analysis software at their disposal allowing for an observer independent objective diagnostic evaluation.

A further limitation of the current gold standard for the diagnosis of CD is due to the possibly patchy distribution of intestinal mucosa areas affected by CD in the midst of normal mucosa [5,62]. If, unfortunately, biopsies are taken only from areas of healthy mucosa within the duodenum the proper diagnosis of CD will be missed due to sampling error. A diagnostic tool based on computer-aided pattern analysis of endoscopic video clips could indicate areas that are damaged by CD thus improving the targeting accuracy of biopsy. Additionally, such a system would allow gastroenterologists who do not routinely take duodenal biopsies recognize mucosal alterations triggering a decision to biopsy. So even in a diagnostic scenario involving biopsies, computer-assisted video analysis contributes to a more reliable diagnosis.

In this work, we try to draw a comprehensive picture of the research focused on computer-assisted diagnosis of CD in endoscopic data. In Section 2 an overview of current endoscopic techniques used in automated diagnosis is given with a discussion of the different properties and requirements for computer-assisted systems. Section 3 provides a general overview of techniques devised for classification of CD. Concepts for handling image degradations in the challenging endoscopic environment are presented in Section 4. Common issues and flaws in the methodical evaluation of techniques and the available ground-truth in medical image classification are covered in Section 5. Finally, Section 6 concludes the paper.

## 2 Endoscopic techniques used in diagnosis of celiac disease

Besides standard upper endoscopy, several new endoscopic approaches for diagnosing CD have been applied [14] and used in research on computer-assisted diagnosis. The modified immersion technique (MIT [7–9]) allows detailed scanning of the mucosal surface for villi. Technically, water is rapidly instilled into the duodenal lumen after evacuation of air by suction through the endoscope. Villi, if present, straighten up in water and appear as tiny finger-like structures. In a detailed evaluation, Hegenbart et al. [52] have presented strong empirical evidence, that MIT is superior for computer-aided diagnosis as compared to the conventional imaging technique.

Narrow band imaging (NBI [30]) uses specific blue (440–460 nm) and green (540–560 nm) wavelengths for illumination to enhance the contrast of vascular patterns on the mucosal surface. This imaging modality could be a promising technique for classification of celiac tissue. Valitutti et al. [90] recently proposed the use of NBI combined with the water immersion technique which could also be explored for automated diagnosis. At this point in time, a systematic assessment of all four imaging modalities in flexible endoscopy for computer-assisted diagnosis is still open.

Confocal laser endomicroscopy (CLE, also known as visual biopsy) is a novel technology allowing real-time in vivo microscopy. CLE allows the inspection of multiple mucosal layers, employing a laser at different focal points and has been shown to be a promising technique for diagnosis of CD in endoscopy [70].

A general drawback of endoscopy using flexible endoscopes is the limited range. As a way of inspecting a much larger area of the intestine, wireless capsule endoscopy (WCE [80]) is used. In WCE, a small capsule equipped with a camera is swallowed by the patient. The capsule records images of the mucosal tissue during its passage through the intestine, which is then analyzed by a clinician or potentially by an automated system for diagnosis.

**Fig. 1:**
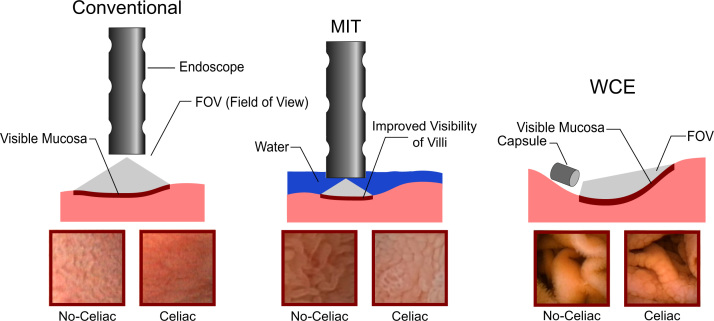
Conventional endoscopic imaging, the modified immersion technique (MIT) as well as wireless capsule endoscopy (WCE).

**Fig. 2:**
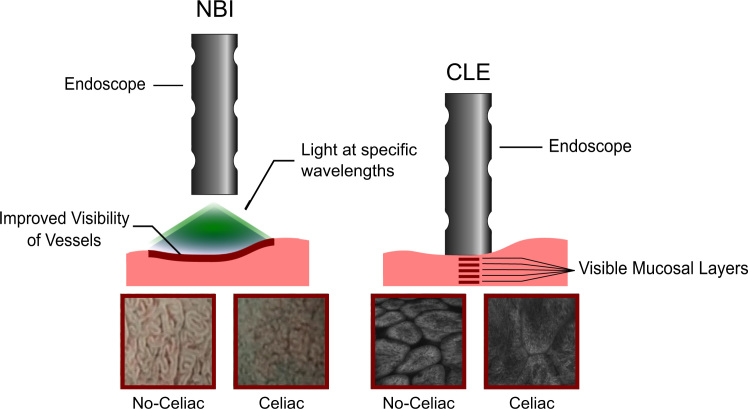
Narrow band imaging (NBI) and confocal laser endomicroscopy (CLE).

Figs. 1 and 2 schematically illustrate the different endoscopic techniques used for computer-assisted diagnosis of CD.

### 2.1 Endoscopic markers and diagnosis of CD

The most prevalent endoscopic markers for CD include scalloped folds, mosaic patterns of the mucosa and a nodular mucosa [25]. After entering the duodenum with the endoscope, the endoscopist searches for visible CD-induced mucosal lesions indicated by these markers. All of this features become more pronounced and easier detectable when using MIT. Consequently, NBI is employed (if available) to specifically delineate the outline of the residual villous structures (if present). NBI is used because images captured using this modality allow a better assessment of the villous height and shape compared to the conventional white light endoscopy.

It has been reported that the prevalence of endoscopic markers is significantly lower for partial villous atrophy (58%) than for subtotal or total villous atrophy (82%) [26]. Consequently, systems based on visual endoscopic markers might be subject to error in diagnosis of CD due to missing features even in the high-risk population. CLE is a technique that could potentially be used for classification in accordance to the histologically described changes of the mucosal tissue instead of relying on visual endoscopic markers with low sensitivity.

Effort has been put into the identification of temporal features in CD such as the variations in brightness and texture [22,16,18,21] as well as changes in the duodenal wall motility [17]. This sort of features were mainly used in WCE and have shown promising results. They have not yet been evaluated in a large clinical study yet.

**Fig. 3:**
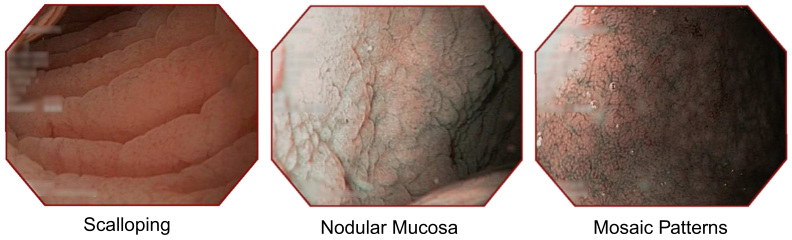
The most prevalent endoscopic markers in CD.

Fig. 3 illustrates the most prevalent endoscopic markers captured during flexible endoscopy.

### 2.2 Characteristics of endoscopic techniques

The natural features of endoscopic techniques used in the diagnosis of CD call for different approaches towards automated diagnosis. Data from flexible endoscopy is characterized by high image resolution but rapid non-monotonic movement. As a consequence, spatio-temporal features are rather inappropriate for this type of data and most work in this context was done using the approach from a more classical texture classification perspective. CLE combines microscopy with standard endoscopy, providing the highest image resolution of all endoscopic methods. Although classic endoscopic degradations are not expected during endomicroscopy, targeting of suspect areas for inspection is a vital part of the diagnosis chain and comparable to flexible endoscopy. Consequently, a significant number of challenges are shared between flexible endoscopy and CLE. The most significant difference of those two techniques compared to WCE is the possibility of interaction with a clinician during the procedure.

Data captured during WCE is characterized by slower, monotonic movement and lower resolutions. This is resulting from the passive propulsion of the camera through the intestine caused by peristalsis. Due to the nature of the data, spatio-temporal features are a much more promising concept as compared to flexible endoscopy and CLE. The missing possibility of interaction and the large amount of recorded data requires different concepts for computer-assisted systems as compared to flexible endoscopy and CLE. In WCE, automated systems focused on reducing the workload of a manual analysis and interpretation by clinicians and providing additional, discriminative statistics for diagnosis seem to be most valuable.

The properties of flexible endoscopy, CLE and WCE are quite divergent. Consequently, it is unlikely that methods developed for one endoscopic technique can be used in the other in a straightforward manner. Table 1 gives a summary of the most relevant properties of each endoscopic methodology for computer-assisted systems.

## 3 Image representations used in automated diagnosis of CD

The nature of data provided by endoscopy requires systems for computer-assisted diagnosis to perform visual analysis. Consequently, the main focus of research found in the literature is focused on methods for the visual classification of duodenal tissue.

### 3.1 Materials used in experimental studies

The unconstrained gastrointestinal environment provides a challenging scenario for visual analysis. Endoscopic image degradations such as blur, bubbles, specular reflections, non-opaque fluids as well as rapidly changing viewpoints and camera-distances complicate a systematic analysis of developed methodologies. To avoid unwanted side-effects caused by such degradations, current research focused on the visual classification of intestinal mucosa affected by CD is based on a defined set of (possibly unrealistic) constraints. Due to the lack of publicly available data-sets experimental evaluation and development is performed using specific private data-sets.

***WCE***: The data used during experimentation and consequently all reported results in WCE [22] are based on a rather small number of patients (approximately 20). The sequences were captured in a follow up examination after the diagnosis of CD had been confirmed using standard flexible endoscopy including biopsies. All patients with positive histology (Marsh–Oberhuber) and serology were on a GFD. The authors report, that these patients are still considered to have active CD due to the fact that a period of multiple months is needed for the diet to cause a reduction in small intestinal villous atrophy. The entire sequences were interpreted by three experienced gastroenterologists and selected video clips with 200 image frames in length were exported as 576×576 pixel images.

***Flexible endoscopy***: The data used during experimentation in flexible endoscopy varied between publications. Initial work [95,94] was based on a rather small amount of data (approximately 200 images of the duodenal bulb and approximately 200 images of the second part of the duodenum) recorded with the conventional image capturing technique. Hegenbart et al. [52] compared MIT with the conventional technique and found MIT to be superior for computer-assisted diagnosis. Subsequently, all work in flexible endoscopy was based on images captured using MIT. The amount of data that was used was reported as 600 images from 170 patients in 2011 [93,56] and 1050 images with 320 patients in 2014 [67]. The results reported in this section are all based on comparable data-sets from 2011.

The flexible endoscopy data-set is based on video data captured during standard endoscopy (the majority using the MIT) in children suffering from signs and symptoms making upper endoscopy necessary. The severity of the disease was classified based on histological findings (Marsh–Oberhuber) in children with positive CD serology. During the procedure, still images of areas with specific visual manifestations of CD or the absence of the disease were captured by the endoscopist. The full image frames were later manually segmented by a domain expert into possibly multiple, non-overlapping regions of size 128×128 pixels [52].

***CLE***: The CLE data-set used by Grisan et al. [45] in their initial work is still of rather small size (128 images from 30 patients). Subjects were recruited from a population with known CD, suspected CD and controls. CLE images and forceps biopsies of the same site were then taken sequentially at standardized locations.

A variety of different classification methodologies, including variations of support vector machines (SVM), *k*-nearest neighbor classifiers (*k*NN), Bayes classifiers and random forests were used in this context. According to the published literature, it seems clear that the most significant impact on the accuracy of automated diagnosis lies within the proposed image representations however. Consequently, the focus of the presented methodologies in the following subsections lies on the feature extraction methods used for classification of CD. Due to different data-sets and evaluation methodologies used across the literature, it is difficult to compare the obtained results. Consequently, herein we will only report the results of methods that were evaluated in a comparable fashion. The presented experiments were based on the data described in this section. Note that the data-sets used in experimentation was approximately balanced between cases and controls (in terms of samples) in this experiments. For the sake of consistency we present the classification accuracy (denoted as overall classification rate (OCR)) of each paper in the following section. Table 2 presents a summary of the reported results.

### 3.2 Spatial domain features

In flexible endoscopy, special attention was payed to image representations in the spatial-domain. A method particularly developed for classification of CD is the Shape-Curvature-Histogram (SCH) [34,36] (OCR 85–87) which is a robust shape based feature, describing local curvature along edges and is supposed to capture the shape of duodenal villi.

Ciaccio [15] explicitly measured the fissure length in endoscopic images in a semi-automatic system (OCR 64). Based on manually selected sub-images, morphological skeletonization was performed. The total length of the skeletonized fissures was then used as one-dimensional feature.

Other, more general purpose image representations that were applied include the Edge Co-Occurrence Matrix [81,34] (OCR 86) describing edge information, the classical Haralick features [51,34] computed from gray-level Co-Occurrence Matrices (OCR 87) and features based on the autocorrelation between an image and a morphologically transformed version of the same image using the Distribution of Spatial Size (SSD) [2,34] (OCR 90).

A very promising class of spatial image representations was based on Local Binary Patterns (LBP [78]). LBP represents images as distributions of pixel neighborhoods, encoded as binary patterns and has been shown [55,53,58,41,43,45] to be very robust and accurate in classification of CD (OCR 83).

Various specialized LBP-variations have been developed for computer-assisted diagnosis of CD. Vécsei et al. [93] proposed two new LBP-based methodologies, particularly optimized for endoscopic environments. The WT-LBP (OCR 88) method was designed to combine different wavelet subbands with appropriate LBP-based operators, while the ELTP (OCR 86) method combines the benefits of the robust Local Ternary Patterns (LTP [86]) with the highly discriminative Extended Local Binary Patterns (ELBP [63]) using an adaptive thresholding, based on local image statistics.

Explicit frequency filtering using appropriate cut-off frequencies in combination with LBP-based image representations [53] has been explored (OCR 83-86) as well. Gadermayr et al. [41] employ LBP based on an adaptive neighborhood for implicit distortion correction while Hegenbart et al. [58] compute a scale- and orientation-adaptive LBP-representation, providing reliable features in scenarios with highly varying orientations and scales. Finally, Grisan et al. [45] used LBP with a pyramidal image decomposition of CLE imagery (OCR 93).

Spatial domain features seem to be very well suited for the visual classification of CD. In addition to providing a high classification accuracy, the majority of methods in this category are robust in terms of distortions and fast to compute. Both of these strengths are necessary requirements for real-time applications in endoscopy.

### 3.3 Transform domain features

Various feature extraction methods used for classification of CD in flexible endoscopy are based on image domain transformations. A benefit of computing features in transform domains is the possibility of employing different categories of information, such as multi-scale and multi-orientation information in wavelets subbands or frequency and phase information in the Fourier domain.

Wavelet-based methods have been used frequently for the classification of CD throughout literature. The most elementary features [49,34] are based on classical statistics such as the mean, the standard deviation or entropy of subband-coefficient energies (OCR 70). Due to the low discriminative power of this type of features, more sophisticated approaches based on the wavelet packet decomposition have been employed. Using the best basis centroids method (BBC) [71,95,93,34] (OCR 77–82) as well as the local discriminant basis (LDB) algorithm [49,83,95,93,34] (OCR 79.5–82.5) the discriminative power of elementary statistical features could be further improved.

Other wavelet-based image representations that have been applied include classical Gabor statistics [74,52,93] (OCR 80) as well as features based on Gaussian Markov Random Fields (MRF) in the wavelet domain [48,93,34] (OCR 78.5–80.5).

In addition to the standard wavelet transform, the dual-tree complex wavelet transform (DT-CWT, [84]) has been applied for classification of CD. Discriminative features include correlation signatures [91,93] (OCR 82) as well as the Weibull parameters of the marginal distribution of the complex wavelet coefficients [68,52,93] (OCR 77–82).

In a complementary fashion to the wavelet transform, Fourier based features have been proposed as well. Ring shaped filters with variable width, optimized in an evolutionary framework, were used to compute elementary statistics of the frequency domain coefficients [94,52,93] (OCR 82).

Comparing the classification performance achieved by features computed in transform domains (OCR 70–82.5) with the rates of spatial features (OCR 81–90), a lower accuracy was obtained when using transform domain features. This could potentially be caused by endoscopic distortions, negatively influencing the transformation process or a generally lower discriminative power of the image representations.

### 3.4 Scale-invariant features

Due to highly varying camera-perspectives and scales in endoscopic sequences, image representations invariant to scaling and rotation could be superior to features affected by such transformations. Consequently, scale-invariant image representations have been applied to data from flexible endoscopy [89,60,58]. Among the most promising scale-invariant features are the Multi-Fractal-Spectrum [99,60] (OCR 89) and Local Fractal Features based on MR8 filtering [92,60] (OCR 92). Scale- and Orientation-Adaptive LBP [58] have been specifically designed for environments with highly varying camera-scales and orientations and are another promising image representation for endoscopy.

The majority of scale-invariant methods perform similarly to non-invariant image representations however. This is true for methods based on the DT-CWT and the D^3^T-CWT [89,72] (OCR 66-88), dense SIFT features [79,60,58] (OCR 83.5) as well as Multiscale Blob Features [98,60] (OCR 86).

Among the worst methods in terms of classification accuracy were features based on pulse coupled neural networks such as SCM [100,60] (OCR 64) and ICM [73,60] (OCR 67) as well as slide Matching of Gabor features [33,60] (OCR 71–75) and Local Affine Regions [69,60] (OCR 71).

The highly variable performance of scale-invariant features illustrates that such image representations are not always suited for difficult environments such as in endoscopy. Specular reflections, blur or bubbles are potentially misleading the identification of key-points or the estimation of affine shapes. Even more, the scale-invariance properties of a large number of methods are based on theoretical concepts and assumptions, which rarely hold true in practice. Consequently, the majority of features specifically designed for scale-invariance perform similarly to features affected by scaling. In general, scale-invariant image representations are computationally more demanding as compared to more elementary features. Therefore, the availability of such methods could potentially be restricted to offline scenarios in computer-assisted diagnosis.

### 3.5 Spatio-temporal features

Spatio-temporal features are a powerful tool for analyzing sequences of images or videos such as produced by WCE. The general benefits of this type of image representation are a higher robustness in terms of extraneous endoscopic degradations and the possibility of identifying CD even if the gastrointestinal manifestations of the disease are not visualized directly in the recorded data.

Initial work [16] (OCR 65) on spatio-temporal features, computed from WCE sequences, was based on elementary local statistics (such as pixel brightness and image texture) measured over 10×10 pixel subimages and then averaged for 56×56 subimages per frame. The authors reported spatio-temporal differences in brightness and texture of celiac images as compared to controls.

In celiac patients, gastrointestinal motility abnormalities were reported [87,3], which may result from a diminished number of enterochromaffine cells producing hormones that regulate intestinal motility. Ciaccio et al. [17] use dynamic estimates of wall motility for image classification by characterizing the motility according to frame-to-frame changes in lumen shape and position (OCR 59).

Based on the assumption that the reduced gastrointestinal motility in celiac disease affects peristalsis, Ciaccio et al. [22,18,21] (OCR 71) estimate the dominant period in a sequence of WCE images as the tallest peak in the ensemble average power spectrum, which is then used to compute salient information based on a set of basis images. The results show that celiac images had more detailed texture than controls and exhibited more variation in brightness. In celiacs, correlations existed between greater textural alterations vs. longer dominant periods.

Shape-from-shading is a technique for the reconstruction of the three dimensional structure of an object, based on illumination information. In [20] (OCR 64), shape-from-shading was applied to reconstruct the duodenal structure from WCE images for an analysis of luminal macro-architecture. Based on a syntactical analysis, protrusions were identified and used to compute volumetric statistics. Significant differences of these statistics between celiac patients and controls were reported.

Due to the relatively low accuracy but complementary characteristic of the presented features, a polling protocol for predicting CD in WCE analysis was presented, based on a majority voting scheme using a variety of features [19]. The experiments showed that a combination of complementary, low-discriminative feature can be used to construct an accurate classification system (OCR 88). This is impressive because no manual segmentation of WCE sequences was performed and endoscopic image degradations were handled implicitly. As a consequence of the small number of used sequences during experimentation, however the general performance of this system on independent data is still unclear.

## 4 The gastrointestinal-tract: a challenging environment

The gastrointestinal tract is a very challenging environment for computer-assisted diagnosis. As mentioned in Section 3, the majority of methods for automated diagnosis were developed based on constrained data-sets, allowing systematic and objective evaluations. Obviously, this is not a realistic scenario. As a consequence, the impact of common image degradations as well as concepts for handling challenges provided by the endoscopic environment was specifically studied in a significant number of publications.

The appearance of duodenal tissue is highly dependent on extrinsic camera parameters such as distance and angle towards the mucosa. As a combined effect of the geometric properties of the gastrointestinal tract with the large field of view of optical systems used in endoscopic hardware, certain areas of the visualized tissue are generally blurred or underexposed and noisy. The insufflation and suction of air during flexible endoscopy as well as the instillation of water into the lumen can lead to a significant amount of bubbles on the duodenal walls. Fluids on the mucosal surface can potentially cause specular reflections of the illuminating point light sources attached to the endoscope. These types of image degradations are likely to affect the feature extraction process and the performance of a computer-assisted system for diagnosis.

Due to the challenging environment, methods for informative frame identification and segmentation are generally applied across the literature in related fields of endoscopic image processing. Most of the proposed methodologies, however, are tailored toward specific applications or endoscopic scenarios and cannot be used in a straightforward manner for CD diagnosis.

An approach combining informative frame detection and segmentation in classification of CD was proposed by Gadermayr et al. [42]. The authors combine multiple elementary quality measures (brightness, contrast, blur, noise and reflections) to perform an automated segmentation of image frames from flexible endoscopy.

An alternative concept by Hegenbart et al. [54,57] was based on a one-class support vector machine (SVM), solely trained on celiac features for implicit handling of certain endoscopic image degradations.

In WCE, a way of handling extraneous features is based on robust spatio-temporal features computed from noise insensitive basis images [18,21].

### 4.1 The duodenal gang: blur, bubble, noise and reflection

**Fig. 4:**
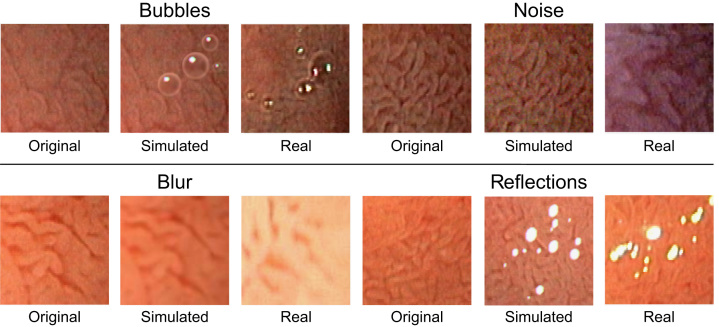
Real and simulated endoscopic image degradations.

It is safe to assume that endoscopic image degradations have a negative effect on the performance of computer-assisted diagnosis. The severity of these effects is unclear however. Consequently, Hegenbart et al. [54] systematically investigated the implicit handling of the most common image degradations (blur, bubble, noise and reflections) based on a one-class SVM. In an experimental evaluation using LBP-based methods for feature extraction, image degradations were simulated to allow a fine grained analysis (with corresponding ground-truth) of the individual effects on the proposed classification pipeline. Fig. 4 illustrates real and simulated endoscopic image degradations used by Hegenbart et al. [54].

The authors report that certain types of image degradations, such as bubbles and reflections, only affect a subset of LBP-based methods and can actually be compensated using the proposed approach. Blur and noise had the most impact on the accuracy of the system. The unconstrained classification of CD based on LBP using a one-class SVM is feasible to some degree. Extreme cases of image distortions might require an additional step of informative frame identification however. By relaxing the needs for informative frame identification to extreme cases, the general reliability of a fully automated system could possibly be increased.

Another, unorthodox approach is based on degradation adaptive texture classification [39]. Using a set of measures for the severity of image degradation (noise, blur, scale, illumination and contrast), the set of features used for training is adaptively adjusted in such a way that only images with a high similarity in terms of distortions to the image classified are used. The authors reported significantly improved classification accuracies (up to 5 percentage points) as compared to using the entire corpus of training data.

### 4.2 Gastrointestinal regions: Where am I?

Sequences in flexible endoscopy are commonly non-monotonic in terms of the passage through gastrointestinal regions. During the procedure, the endoscopic tip often traverses the stomach and the duodenum multiple times. Consequently, the visual appearance of esophageal and gastric tissue in endoscopic data is a common thing. A classification model based on duodenal tissue is potentially subject to error if visualized tissue of other (unknown) gastrointestinal regions is provided. Consequently, Hegenbart et al. [57] studied if tissue visualized from other gastrointestinal regions (esophagus and stomach) would be misclassified as CD using their approach based on a one-class SVM.

**Fig. 5:**
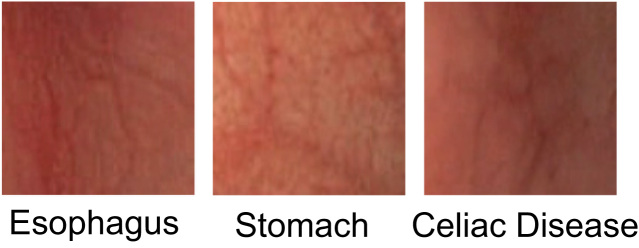
Visual appearance of different GI-regions.

The authors reported that the visualized appearance of esophageal and gastric tissue can be very close to duodenal tissue affected by villous atrophy (see Fig. 5). The experimental evaluation indicates that celiac tissue cannot be distinguished from esophageal and gastric tissue in such a classification approach. Consequently, it is likely that an additional mechanism for tracking the endoscope׳s location or identification of the intestinal region is required to handle non-monotonic transits and the visualization of unknown gastrointestinal regions in a fully automated system.

### 4.3 Extrinsic camera parameters: high variance

**Fig. 6:**
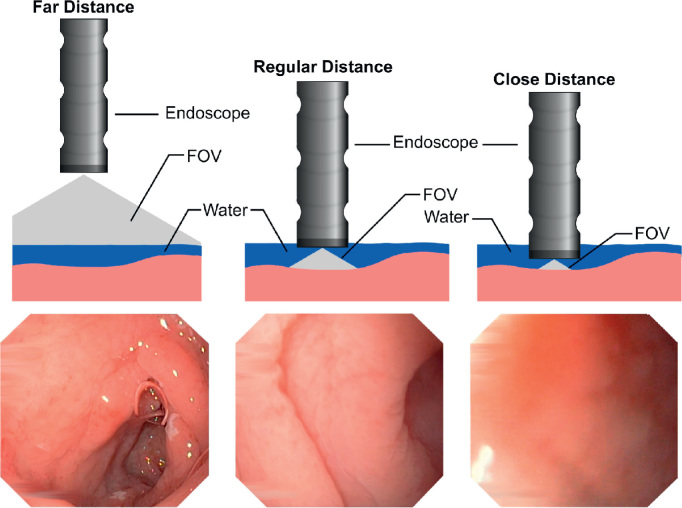
Visualization of tissue at different camera distances.

Highly varying extrinsic camera parameters such as distance and angle to the mucosa can potentially lead to a blurred visualization of the tissue (close distance) or to a missing visualization of small spatial structures such as villi (far distance) and could therefore be inappropriate for visual classification (see Fig. 6).

The effects of varying camera-scales were explicitly studied by Hegenbart et al. [57]. Experimental data indicated that the visualization of duodenal tissue at close and far distances is inappropriate for classification with features affected by scaling such as LBP.

Consequently, scale- and viewpoint-invariant methods have been employed [60] for the classification of CD. In this work, the authors showed in a comprehensive study that scale-invariant image representations often exhibit an intrinsically lower discriminative power as compared to features affected by scaling. Consequently, scale-invariant features often do not pose a significant benefit. A small subset of such methods (Fractal Dimension and affine-adaptive LBP) were promising however.

Recently, a scale- and rotation-invariant image representation based on highly discriminative LBP (SOA-LBP) has been proposed, particularly for scenarios with significantly varying camera-scales [58] such as endoscopy.

### 4.4 Interlacing

Interlaced scanning is a technique that has been widely used in video systems to double the perceived frame rate without increasing the required bandwidth. This technique is still in use by certain endoscopic video hardware today. Various specialized de-interlacing techniques have been developed over the last decades to re-construct full frames from two interlaced half-frames. The impact of interlaced scanning and the benefits of applying suited de-interlacing techniques for computer-assisted systems was specifically studied [59].

The authors state that de-interlacing does not have a significant positive effect on the classification accuracy of endoscopic data with indication for CD. The benefits of applying de-interlacing were comparable to the effects of Gaussian filtering. Considering the accuracy of the system, the authors could not identify significant differences between simple and more complex de-interlacing techniques. Consequently, computer-assisted systems based on interlaced data could potentially be used without an explicit need for de-interlacing.

### 4.5 Do not believe your eyes: lens distortion

**Fig. 7:**
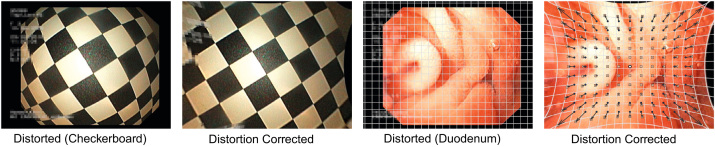
Lens distortion and distortion correction applied to endoscopic images.

A special type of endoscopic image degradation, present in all endoscopic images, is a consequence of the wide-angle nature of the optical systems used in endoscopic hardware. Such barrel-type distortions introduce non-linear changes in the images, leading to a significantly smaller visualization of outer areas. Fig. 7 illustrates the visual manifestation of lens distortions in endoscopic imagery.

The effects of endoscopic lens distortions as well as distortion correction on computer-assisted diagnosis have been extensively studied [46,34,43,50]. The general consensus in the literature is that the effects of lens distortion and the benefits of distortion correction are highly dependent on the used type of features. Features based on low-frequency components of the images are merely affected by lens distortion while image representations based on high-frequency components clearly benefit from distortion correction. The largest improvements reported were in the range of 5–6 percentage points. In some cases the classification accuracy dropped by approximately 9 percentage points however.

Interpolation artifacts and varying camera-scales were identified as the main restrictions of distortion correction performance [35]. Consequently, distortion adaptive classification [38,40], distortion compensated features based on the Fourier transform [37] as well as intrinsic distortion correction [41] have been proposed.

A particular application for lens distortion correction is an improved visualization for clinicians during endoscopy [43]. It was shown in a study involving several gastroenterologists that the accuracy of diagnosis by domain experts using such a system is actually negatively affected. This is most likely a consequence of their medical training based on distorted imagery.

## 5 Evaluation of methods in medical image classification

The nature of medical data differs from the characteristics of data used in more classical texture classification scenarios. As a consequence of a potentially small number of available patients and an uneven distributions of healthy and unhealthy individuals undergoing treatment, multiple samples per patient are often used to build balanced data sets of sufficient size. This often results in an intrinsic bias of data sets used in medical image classification.

To deal with restricted amounts of data available for experimentation, cross-validation techniques are used for predicting how well developed methodologies will generalize. Particularly in medical image classification, assumptions of such techniques are often violated by the inherent intrinsic bias of the created data-sets. The performance predicted by cross-validation is consequently subject to error.

Methods for feature extraction or classification are usually parametrized by a set of values such as thresholds, number of neighbors or filter coefficients. The optimal range of values of such parameters is often unclear from a theoretical standpoint and usually determined empirically. Consequently, parameter optimization is often performed in the process of development or evaluation. In a combination with cross-validation, optimization can be dangerous and can potentially lead to over-fitting, reducing the accuracy of performance prediction significantly.

A common flaw in the evaluation of methods is based on using the predicted classification accuracy of the cross-validation as the objective function for optimization. Hegenbart et al. [56] reported prediction errors when using this strategy of up to 7.5 percentage points (OCR 92 vs. OCR 84.5). The authors suggest to use nested cross-validations to avoid such errors, optimizing parameters in a separate cross-validation based on the current training fold.

Due to violated assumptions of cross-validation techniques, caused by the intrinsic bias of medical image sets, errors of up to 7 percentage points were reported (Leave-One-Out Cross Validation: OCR 90; Distinct Sets: OCR 83). The violation of assumptions in cross-validation techniques can be avoided by using a data partition based on patients instead of images.

The prediction error of nested-cross validations using patient-based partitions was reported to be only 1.4 percentage points as compared to the most realistic scenarios with two independent data-sets. Although nested-cross validations add a significant amount of computational complexity to the experimental setup, such schemes are mandatory for realistic predictions if optimization is performed.

### 5.1 Staging schemes and noisy ground-truths

In a revision of the Marsh-scheme for staging the severity of CD, Oberhuber et al. [77] classified Marsh type 3 lesions into three sub-groups. Unfortunately, the definitions of type Marsh-3A (mild or moderate villous shortening) and Marsh-3B (marked villous shortening) lack objective criteria, contributing to an increased intra- and inter-observer variability [31,76,1].

Only four publications [93,60,15,43] on computer-assisted diagnosis of CD are focused on a reduced 4-class Marsh-like scenario (including classes Marsh-0, Marsh-3A, Marsh-3B and Marsh-3C). The authors reported classification rates between 50 and 68% and point out problems with lacking distinction of visual appearance between classes of type Marsh-3. Vécsei et al. [93] suggest to use more simplified, visually focused systems such as proposed by Corazza and Villanaci [24] or Ensari [31] for automated diagnosis. Consequently, the majority of work throughout the literature has been focused on the clinically most relevant two-class case (celiac disease vs. healthy).

As a side-effect of the potentially high intra- and inter-observer variability, the histological Marsh–Oberhuber based ground-truth used for developing computer-assisted systems is subject to a certain amount of noise. As a consequence of the patchy distribution of CD, discrepancies between areas with known histology and images used for developing computer-assisted systems could potentially exist.

A noisy ground-truth as basis for development and training of a computer-based system could be problematic and effectively reduce the accuracy of and confidence in the automated diagnosis. Recently, Kwitt et al. [67] were able to show that existing noise in ground-truths can be compensated by a sufficiently large corpus of training data. In a crowd-sourcing context, endoscopic data with indication for CD were labeled by non-experts and used to create a classification system that performed comparably to a system trained on data with histological ground-truth (and therefore a substantial smaller amount of label noise). This finding consequently indicates that a moderate amount of label noise (as present in histological ground-truths) can actually be compensated implicitly by a classification system based on a training corpus of sufficiently large size.

## 6 Conclusion

Since the first publication on computer-assisted diagnosis of CD in 2008, a lot of progress towards a fully automated system has been made. A large variety of image representations has been studied and a solid knowledge of the behavior of methods in an endoscopic environments has been acquired. Based on constrained data-sets from flexible endoscopy, CD can currently be classified with an accuracy of up to 92%. Based on a majority voting using multiple complementary spatio-temporal features, classification accuracies in sequences of WCE currently reach 88%. The small number of sequences used for evaluation, however, still leaves open questions about the general performance of this methodology. Recently reported results by Grisan et al. using data from CLE indicate that this imaging modality could potentially be promising for automated diagnosis (OCR 93). Again, the small data-set size in CLE is problematic.

Significant effort was put into the study and development of concepts for handling various challenges in endoscopic imagery such as image degradations, heavily varying extrinsic camera-parameters, interlacing and lens distortion. As an alternative approach to informative frame detection, a classification pipeline based on a one-class SVM has been proposed for the implicit handling of the most common endoscopic image degradations.

Robust features computed from degradation insensitive basis images have been used to implicitly handle extraneous features in WCE and in an unorthodox approach, degradation adaptive classification has been successfully performed.

Lens distortion has been shown to affect the accuracy of automated diagnosis. Consequently, distortion adaptive classification, distortion compensated features as well as intrinsic distortion correction have been used to improve the accuracy of classification.

A solid understanding of issues and common flaws in the evaluation of methods in a medical context has been gained. It was shown that medical data in particular has to be treated with extreme care if cross-validation is used in combination with any type of parameter optimization. Empirical data indicates that a moderate amount of label noise in the ground-truth can be compensated if a large amount of training data is available.

Although a big step towards the establishment of a fully automated system has been made, there are still a lot of challenges to be solved before a system for computer-assisted diagnosis can be used in clinical routine. One of the most stressing issues, especially in flexible endoscopy and CLE, is the development and establishment of robust and accurate methodologies for informative frame detection and segmentation. Although the implicit handling of various endoscopic image degradations is possible to some degree, images affected by blur and large camera distances are not very well handled in such an approach.

Combining spatio-temporal features with highly discriminative texture features as used in still image classification is another challenging but promising concept. It may be possible that other highly significant celiac markers could be integrated into such an approach, potentially improving the general accuracy and robustness of the systems.

Finally, it should be a general ambition to provide publicly available data-sets of CD. We are highly convinced that the research progress and interest on computer-assisted diagnosis of CD would thrive if public data-sets were available to fellow researchers.

## Conflict of interest statement

None declared.

## Figures and Tables

**Table 1:** Relevant properties of endoscopic techniques for computer-assisted diagnosis.

**Property**	**Flexible endoscopy**	**WCE**	**CLE**
Movement	Fast	Slow	None
Resolution	High	Low	High
Interactive	Yes	No	Yes
Modalities	Multiple	Single	Single
Field of view	120–170°	140–170°	500×500μm

**Table 2:** Accuracies (OCR) of feature representations used in automated diagnosis of CD.

**Flexible endoscopy**			
Spatial Size Distribution	90	LDB	79.5–82.5
Wavelet-based LBP	88	MRF	78.5–80.5
Local Binary Patterns	83–86	Correlation signatures	82
Shape-Curvature-Histogram	85–87	Fourier statistics	82
Local Fractal Dimension - MR8	92	Multi-Fractal-Spectrum	89
Multiscale Blob Features	86	Dense SIFT	83.5
	**WCE**		

Basis Image Statistics	71	Local Image Statistics	65
Volumetric statistics	64	Wall Motility	59
	**CLE**		

Pyramidal LBP	93		
